# Serum Uric Acid as a Potential Risk Marker for Atherosclerotic Cardiovascular Disease in Japanese Adults: Using the Hisayama Study-based Prediction Model

**DOI:** 10.31662/jmaj.2025-0129

**Published:** 2025-08-01

**Authors:** Takashi Matsuyama, Mito Yamawaki, Jun Furuya, Kyosuke Omata, Mai Kaneko, Ryuichi Furuya, Hideo Yasuda, Hirotaka Fukasawa

**Affiliations:** 1 Renal Division, Department of Internal Medicine, Iwata City Hospital, Iwata, Japan; 2 First Department of Medicine, Hamamatsu University School of Medicine, Hamamatsu, Japan

**Keywords:** atherosclerosis, cardiovascular disease, predictive markers, serum uric acid, the Hisayama study

## Abstract

**Introduction:**

Atherosclerotic cardiovascular disease (ASCVD) risk profiles vary among populations. In Japan, a prediction model for the 10-year estimate of ASCVD risk, integrating coronary artery disease and atherothrombotic cerebral infarct risk assessments, has been developed based on the Hisayama Study. While hyperuricemia can promote atherosclerosis, serum uric acid levels are not included as a risk factor in the 10-year ASCVD prediction model.

**Methods:**

We investigated the association between serum uric acid levels and 10-year ASCVD risk in 5,984 Japanese adults (3,285 males, 2,699 females) aged 40-79 years who underwent health check-ups between 2020 and 2022. The 10-year ASCVD risk scores were calculated using the aforementioned prediction model.

**Results:**

Participants in the lowest quartile of serum uric acid levels (3.8 ± 0.6 mg/dL) had a mean 10-year ASCVD risk of 2.1 ± 3.2%, while those in the highest quartile (7.2 ± 0.7 mg/dL) had a mean risk of 4.3 ± 4.6% (p < 0.001). A significant positive correlation was observed between serum uric acid levels and 10-year ASCVD risk scores (r = 0.34, p < 0.001). Multiple linear regression analysis also revealed that serum uric acid levels remained an independent predictor of 10-year ASCVD risk after adjusting for other risk factors (β = 0.02, p < 0.001).

**Conclusions:**

This study indicates that serum uric acid levels may serve as a useful marker for ASCVD risk assessment, particularly in the Japanese population.

## Introduction

Atherosclerotic diseases, including coronary artery disease and atherothrombotic stroke, are a group of conditions characterized by atherosclerosis. Due to the significant mortality rates associated with these diseases, prediction and prevention strategies are crucial ^(1)^.

In the United States and Europe, models predicting the 10-year probability of developing atherosclerotic cardiovascular disease (ASCVD) are used, targeting outcomes that combine coronary artery disease and stroke for risk assessment ^(2), (3)^. On the other hand, the Suita Score ^(4)^ was previously used in Japan to predict the 10-year probability of developing coronary artery disease. However, the score was limited to coronary artery disease alone. Therefore, the new guidelines recommend using the Hisayama Study score ^(5)^, which combines risk assessment for both coronary artery disease and stroke.

It is also important to note that cardiovascular risk profiles can vary among populations. In fact, the Japanese population exhibits a distinct pattern characterized by a lower incidence of coronary artery disease but a higher incidence of stroke compared to Western populations ^(6), (7), (8)^. These epidemiological differences underscore the need for population-specific risk assessment scores.

Several studies suggest that serum uric acid (UA) levels are associated with cardiovascular risk factors such as hypertension, insulin resistance, obesity, and hyperlipidemia ^(9), (10)^. However, serum UA levels are not included as a risk factor for ASCVD development in the Hisayama Study score. While some reports suggest that hyperuricemia is an independent risk factor for coronary artery disease ^(11), (12), (13)^, others argue that it is merely confounded by other atherosclerotic risk factors ^(14), (15), (16), (17)^, leading to inconsistent views. Therefore, the causal relationship between serum UA levels and the development of ASCVD remains controversial.

The purpose of this study was to investigate the association between serum UA levels and ASCVD risk in a large Japanese cohort. For this purpose, we utilize the recently developed Hisayama Study-based prediction model, which is tailored for the Japanese population ^(5)^.

## Materials and Methods

### Study design and participants

This was a single-center, retrospective cohort study designed to evaluate the association between serum UA levels and ASCVD risk in the Japanese population.

We recruited individuals who underwent at least one annual health check-up between 2020 and 2022 at the medical check-up center in Iwata City Hospital. Then, a total of 5,984 participants (3,285 males and 2,699 females) aged 40 to 79 years were enrolled in the study. We excluded individuals with a history of ASCVD based on preliminary questionnaires. The requirement for individual informed consent was waived by the ethics committee due to the retrospective nature of the study. Instead, an opt-out approach was employed. Information about the study, including its purpose, procedures, and the participants’ right to opt out, was available through the disclosure document on the hospital’s website. Participants were given the opportunity to opt out of the study if they wished. This study was approved by the Ethics Committee of Iwata City Hospital (No. 2024-003) and adhered to the principles of the Declaration of Helsinki. This study was also registered with the Clinical Trial Registry of the University Hospital Medical Information Network (http://www.umin.ac.jp/, study number: UMIN000056100).

### Data collection and measurements

Participants underwent comprehensive physical examinations, including anthropometric and blood pressure measurements. Blood samples were collected in the morning after an overnight fast. For participants with multiple visits during the study period, data from their most recent examination were used. Laboratory analyses were performed using standard laboratory techniques with various autoanalyzers. Complete blood counts, including hemoglobin levels, were measured using the Sysmex XN-3100 automated hematology analyzer (Sysmex Corporation, Kobe, Japan). Lipid profiles (total cholesterol, high-density lipoprotein [HDL] cholesterol, low-density lipoprotein [LDL] cholesterol, and triglycerides), as well as serum creatinine and serum UA levels, were analyzed using the BioMajesty JCA-BM6700G automated analyzer (JEOL Ltd., Tokyo, Japan). Fasting glucose levels were measured with the GA09IIα glucose analyzer (A&T Corporation, Yokohama, Japan). Hemoglobin A1c (HbA1c) values were determined using the HLC-723G11 automated glycohemoglobin analyzer (Tosoh Corporation, Tokyo, Japan). Proteinuria was assessed using dipstick urinalysis. We calculated the estimated glomerular filtration rate (eGFR) using the Japanese eGFR formula ^(18)^. Information on drinking habits, smoking habits, and exercise routines was collected through self-reported questionnaires.

### ASCVD risk calculation

We calculated the 10-year probability of developing ASCVD (10-year ASCVD risk) using the prediction model based on the Hisayama Study ^(9)^. This formula incorporates age, sex, systolic blood pressure, diabetes status, HDL cholesterol, LDL cholesterol, proteinuria, smoking habits, and exercise habits, achieving an area under the curve of 0.786 in validation cohorts ^(5)^. As the Hisayama Study does not specifically account for antihypertensive or lipid-lowering medications, we did not include the use of these treatments in our analyses.

### Statistical analysis

We report results as mean ± standard deviation for continuous variables and as percentages for categorical variables. Due to the non-normal distribution of 10-year ASCVD risk scores, we applied a logarithmic transformation for analysis. Trends across serum UA quartiles were analyzed using the Jonckheere-Terpstra test. Correlations between 10-year ASCVD risk scores and other parameters were evaluated using Pearson’s product-moment correlation coefficients.

To evaluate the independent association between serum UA levels and ASCVD risk, we conducted multiple linear regression analyses (overall, male, and female). The model adjusted for traditional ASCVD risk factors (age, sex in overall analysis, smoking status, systolic blood pressure, LDL cholesterol, eGFR, HbA1c) as well as hemoglobin levels due to their significant correlation with UA. We additionally adjusted for alcohol consumption status to account for its confounding effects on UA metabolism. We confirmed that there was no significant multicollinearity among the variables in the regression models, with variance inflation factors remaining below 2.1 for all variables. Body mass index (BMI) and waist circumference were excluded from the adjustment variables to avoid overadjustment.

We considered a p < 0.05 as statistically significant. All statistical analyses were performed using the Statistical Package for the Social Sciences software program (version 26, IBM, Tokyo, Japan).

## Results

### Participant characteristics and clinical parameters

This retrospective cohort study enrolled 5,984 participants (3,285 males and 2,699 females) with a mean age of 56.0 ± 10.6 years. Significant sex differences were observed in most clinical parameters (Table 1). Males exhibited higher levels of BMI, waist circumference, systolic blood pressure, hemoglobin, triglycerides, serum creatinine, fasting blood glucose, and HbA1c. Males also showed a higher prevalence of smoking and regular exercise habits. Mean serum UA levels and the calculated 10-year ASCVD risk scores were significantly higher in males compared to females (6.0 ± 1.2 mg/dL vs. 4.7 ± 1.1 mg/dL and 5.0 ± 5.1% vs. 1.4 ± 1.5%, p < 0.001, respectively). On the other hand, females had higher total cholesterol, HDL cholesterol, and eGFR levels. The proportion of current drinkers was higher among females than males. Only mean LDL cholesterol levels did not show a sex difference.

**Table 1.** Clinical Characteristics and Laboratory Parameters of Study Participants.

	Overall	Male	Female	p
	n=5,984	n=3,285	n=2,699	male vs female
Age, years	56.0 ± 10.6	56.6 ± 10.8	55.3 ± 10.4	<0.001
BMI, kg/m^2^	22.9 ± 3.7	23.6 ± 3.5	22.0 ± 3.8	<0.001
Waist circumference, cm	82.0 ± 10.1	84.6 ± 9.5	78.7 ± 9.9	<0.001
Current drinker, %	3,083 (54.8%)	1,229 (37.4%)	1,854 (68.6%)	<0.001
Current smoker, %	1,007 (16.8%)	867 (26.4%)	140 (5.2%)	<0.001
Regular exercise, %	1,840 (30.7%)	1,180 (35.9%)	660 (24.5%)	<0.001
Systolic BP, mmHg	124.3 ± 14.9	126.4 ± 14.0	121.8 ± 15.5	<0.001
Hemoglobin, g/dL	14.0 ±1.5	14.8 ± 1.2	12.9 ± 1.2	<0.001
Total cholesterol, mg/dL	207.2 ± 33.7	202.1 ± 32.2	213.5 ± 34.5	<0.001
Triglyceride, mg/dL	101.0 ± 74.1	113.8 ± 75.2	85.4 ± 69.7	<0.001
HDL cholesterol, mg/dL	63.9 ± 16.0	58.5 ± 14.5	70.4 ± 15.4	<0.001
LDL cholesterol, mg/dL	122.5 ± 29.5	122.3 ± 29.6	122.8 ± 29.5	0.518
Serum creatinine, mg/dL	0.82 ± 0.33	0.93 ± 0.40	0.68 ± 0.14	<0.001
eGFR, mL/min/1.73m^2^	70.1 ± 12.7	69.2 ± 12.8	71.3 ± 12.5	<0.001
Uric acid, mg/dL	5.4 ± 1.3	6.0 ± 1.2	4.7 ± 1.1	<0.001
Proteinuria, %	78 (1.3%)	57 (1.8%)	17 (0.8%)	<0.001
Fasting blood glucose, mg/dL	103.4 ± 18.4	106.3 ± 19.6	99.8 ± 16.2	<0.001
HbA1c, %	5.8 ± 0.6	5.8 ± 0.7	5.7 ± 0.5	<0.001
10-year ASCVD risk, %	3.4 ± 4.3	5.0 ± 5.1	1.4 ± 1.5	<0.001

ASCVD: atherosclerotic cardiovascular disease; BMI: body mass index; BP: blood pressure; eGFR: estimated glomerular filtration rate; HbA1c: hemoglobin A1c; HDL: high-density lipoprotein; LDL: low-density lipoprotein.

### Stratification of ASCVD risk by serum UA quartiles

To investigate the relationship between serum UA levels and ASCVD risk, participants were subsequently stratified into quartiles based on their UA levels (Table 2). The quartile ranges were defined as follows: ≤4.5 mg/dL, 4.6-5.3 mg/dL, 5.4-6.3 mg/dL, and ≥6.4 mg/dL, respectively. As serum UA levels increased across quartiles, a stepwise increase in the mean 10-year ASCVD risk scores was observed, with values of 2.1%, 3.1%, 4.1%, and 4.3%, respectively. Statistical significance was also observed when comparing the scores between the first and fourth quartiles (p < 0.001). Similarly, as serum UA levels increased across quartiles, BMI, systolic blood pressure, hemoglobin levels, LDL cholesterol levels, and serum creatinine levels showed a significant upward trend. Conversely, HDL cholesterol levels showed a significant downward trend as serum UA levels increased. We also conducted another stratified analysis using clinically important cut-off values of serum UA. Since participants with serum UA values of 8.0 mg/dL or higher were rare in this health check-up population, we employed 5.0, 6.0, and 7.0 mg/dL as cut-off values (Table 3). When we stratified by these values, the 10-year ASCVD risk increased as serum UA levels increased (UA < 5.0 mg/dL group: 2.3%, UA 5.0-5.9 mg/dL group: 3.7%, UA ≥ 7.0 mg/dL group: 4.4%, p < 0.05, respectively).

**Table 2.** Clinical Characteristics and 10-Year ASCVD Risk Profiles Stratified by Serum Uric Acid Quartiles.

	Quartile 1	Quartile 2	Quartile 3	Quartile 4	p for Trend
	UA ≤ 4.5 mg/dL	UA 4.6-5.3 mg/dL	UA 5.4-6.3 mg/dL	UA ≥ 6.4 mg/dL	
	n=1,626	n=1,408	n=1,503	n=1,447	
Age, years	54.5 ± 10.5	56.9 ± 10.5	57.2 ± 10.8	55.7 ± 10.6*^#†^	<0.001
Males, %	343 (21.1%)	606 (43.0%)	1,068 (71.1%)	1,268 (87.6%)*^#†^	<0.001
BMI, kg/m^2^	21.4 ± 3.22	22.3 ± 3.4	23.5 ± 3.6	24.5 ± 3.8*^#†^	<0.001
Waist circumference, cm	77.6 ± 9.3	80.3 ± 9.5	83.6 ± 9.4	86.8 ± 9.6*^#†^	<0.001
Current drinker, %	560 (34.4%)	614 (43.6%)	801 (53.3%)	926 (64.0%)*^#†^	<0.001
Current smoker, %	155 (9.5%)	196 (13.9%)	310 (20.6%)	346 (23.9%)*^#†^	<0.001
Regular exercise, %	386 (23.7%)	447 (31.7%)	504 (33.5%)	503 (34.8%)*#	<0.001
Systric BP, mmHg	120.6 ± 15.4	123.6 ± 15.1	125.5 ± 13.9	127.9 ± 13.8*^#†^	<0.001
Hemoglobin, g/dL	13.1 ± 1.5	13.8 ± 1.4	14.3 ± 1.3	14.8 ± 1.3*^#†^	<0.001
Total cholesterol, mg/dL	206.3 ± 34.3	207.9 ± 33.1	205.8 ± 33.3	209.0 ± 34.0	0.141
Triglyceride, mg/dL	80.0 ± 47.4	91.9 ± 54.8	102.4 ± 60.0	132.0 ± 109.5*^#†^	<0.001
HDL cholesterol, mg/dL	69.5 ± 15.3	66.4 ± 16.0	61.4 ± 15.3	57.6 ± 14.9*^#†^	<0.001
LDL cholesterol, mg/dL	118.4 ± 28.3	121.4 ± 28.9	123.8 ± 29.8	126.9 ± 30.6*^#†^	<0.001
Serum creatinine, mg/dL	0.70 ± 0.22	0.77 ± 0.15	0.86 ± 0.26	0.95 ± 0.53*^#†^	<0.001
eGFR, mL/min/1.73m^2^	74.8 ± 12.6	70.7 ± 12.3	68.3 ± 11.9	66.3 ± 12.4*^#†^	<0.001
Uric acid, mg/dL	3.8 ± 0.6	5.0 ± 0.2	5.8 ± 0.3	7.2 ± 0.7*^#†^	<0.001
Proteinuria, %	13 (0.8%)	18 (1.3%)	20 (1.3%)	27 (1.9%)*	0.012
Fasting blood glucose, mg/dL	101.2 ± 21.1	103.1 ± 19.4	104.4 ± 16.4	104.9 ± 15.9*^#^	<0.001
HbA1c, %	5.7 ± 0.7	5.8 ± 0.7	5.8 ± 0.5	5.8 ± 0.5	<0.001
10-year ASCVD risk, %	2.1 ± 3.2	3.1 ± 4.0	4.1 ± 4.7	4.3 ± 4.6*^#†^	<0.001

ASCVD: atherosclerotic cardiovascular disease; BMI: body mass index; BP: blood pressure; eGFR< 0.05 vs. quartile 1 in quartile 4, ^#^p < 0.05 vs. quartile 2 in quartile 4, ^†^p < 0.05 vs. quartile 3 in quartile 4.

**Table 3.** Clinical Characteristics and ASCVD Risk Profiles Stratified by Serum Uric Acid Thresholds (5.0, 6.0, and 7.0 mg/dL).

	Group 1	Group 2	Group 3	Group 4	p for trend
	UA ≤ 4.9 mg/dL	UA 5.0-5.9 mg/dL	UA 6.0-6.9 mg/dL	UA ≥ 7.0 mg/dL	
	n=2,295	n=1,716	n=1,234	n=739	
Age, years	55.1 ± 10.5	57.2 ± 10.7	56.7 ± 10.9	55.2 ± 10.6^#†^	0.003
Males, %	569 (24.8%)	1,308 (60.5%)	1,009 (81.9%)	668 (90.4%)*^#†^	<0.001
BMI, kg/m^2^	21.6 ± 3.3	23.0 ± 3.5	24.0 ± 3.6	25.0 ± 4.0*^#†^	<0.001
Waist circumference, cm	78.1 ± 9.2	82.0 ± 9.6	85.2 ± 9.4	88.1 ± 9.9*^#†^	<0.001
Current drinker, %	830 (36.2%)	844 (49.2%)	741 (60.1%)	486 (65.8%)*^#†^	<0.001
Current smoker, %	234 (10.2%)	324 (18.9%)	256 (20.8%)	193 (26.1%)*^#†^	<0.001
Regular exercise, %	593 (25.8%)	573 (33.4%)	429 (34.8%)	245 (33.1%)*	<0.001
Systric BP, mmHg	121.3 ± 15.4	124.8 ± 14.7	126.8 ± 13.4	128.6 ± 13.9*^#†^	<0.001
Hemoglobin, g/dL	13.3 ± 1.5	14.1 ± 1.3	14.6 ± 1.3	14.8 ± 1.3*^#†^	<0.001
Total cholesterol, mg/dL	207.0 ± 34.0	206.7 ± 33.1	206.2 ± 33.1	210.9 ± 35.3*^#†^	0.125
Triglyceride, mg/dL	82.7 ± 50.8	96.7 ± 53.2	115.6 ± 84.5	143.5 ± 121.5	<0.001
HDL cholesterol, mg/dL	69.0 ± 15.6	63.7 ± 15.8	59.3 ± 14.6	56.3 ± 14.8*^#†^	<0.001
LDL cholesterol, mg/dL	119.0 ± 28.4	122.9 ± 29.3	124.7 ± 30.0	129.0 ± 31.3*^#†^	<0.001
Serum creatinine, mg/dL	0.71 ± 0.20	0.83 ± 0.25	0.90 ± 0.19	1.00 ± 0.72*^#†^	<0.001
eGFR, mL/min/1.73m2	73.8 ± 12.4	69.1 ± 12.5	67.7 ± 11.8	65.2 ± 12.9*^#†^	<0.001
Uric acid, mg/dL	4.1 ± 0.7	5.4 ± 0.3	6.4 ± 0.3	7.7 ± 0.7*^#†^	<0.001
Proteinuria, %	17 (0.7%)	25 (1.5%)	16 (1.3%)	20 (2.7%)*^#†^	<0.001
Fasting blood glucose, mg/dL	101.5 ± 20.4	103.6 ± 17.6	104.8 ± 16.4	105.9 ± 16.6*^#^	<0.001
HbA1c, %	5.7 ± 0.7	5.8 ± 0.6	5.8 ± 0.5	5.8 ± 0.6^†^	<0.001
10-year ASCVD risk, %	2.3 ± 3.3	3.7 ± 4.6	4.3 ± 4.8	4.4 ± 4.4*^#^	<0.001

ASCVD: atherosclerotic cardiovascular disease; BMI: body mass index; BP: blood pressure; eGFR: estimated glomerular filtration rate; HbA1c: hemoglobin A1c; HDL: high-density lipoprotein; LDL: low-density lipoprotein; UA: uric acid. *p < 0.05 vs. group 1 in group 4, ^#^p < 0.05 vs. group 2 in group 4, ^†^p < 0.05 vs. group 3 in group 4.

### Correlation analysis and multivariate regression models

**Figure 1.**
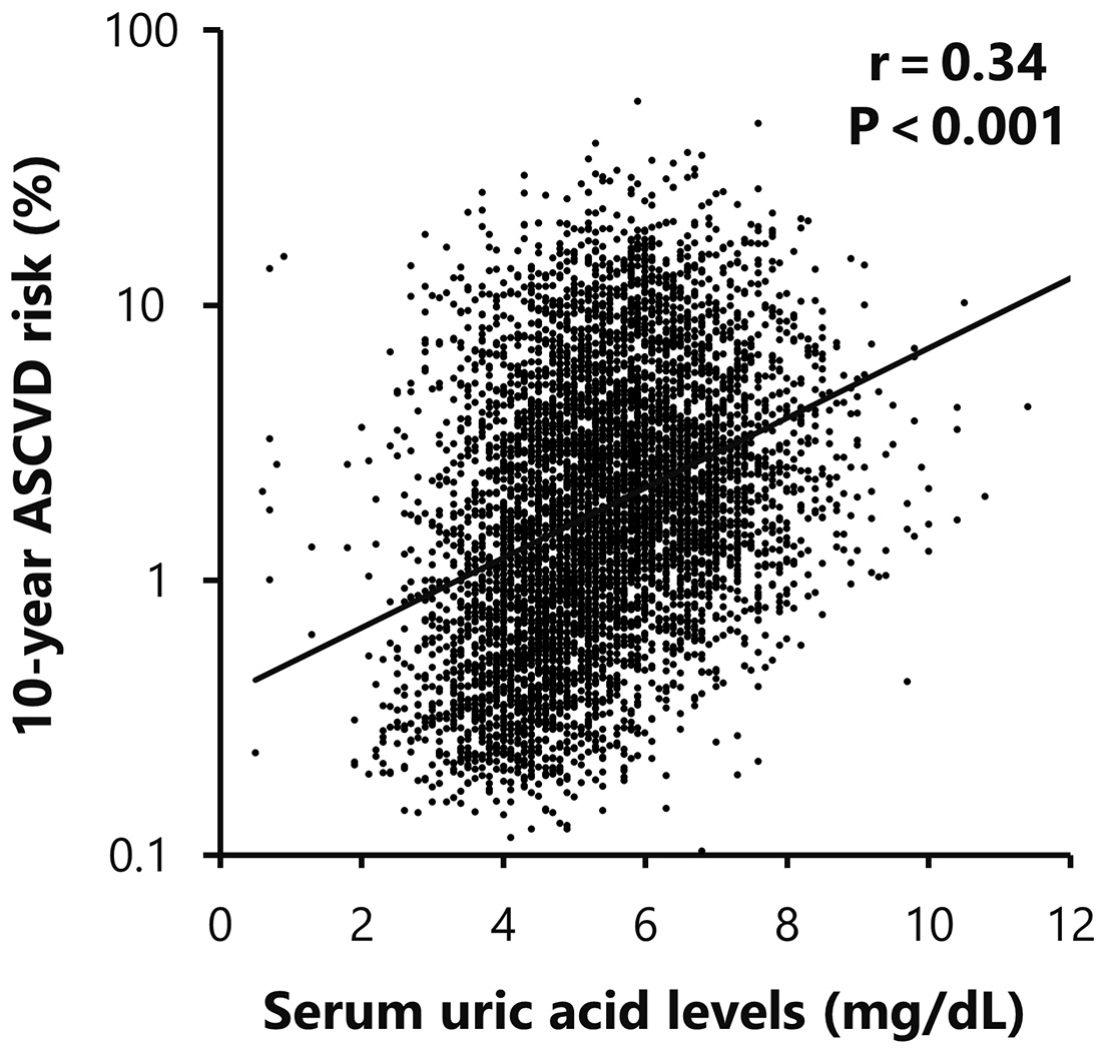
Correlation between serum uric acid levels and logarithmic 10-year ASCVD risk scores. The significant positive correlation was observed between serum uric acid levels and logarithmic 10-year ASCVD risk scores in study participants (p < 0.001). ASCVD: atherosclerotic cardiovascular disease.

The 10-year ASCVD risk showed significant correlations with traditional cardiovascular risk parameters, including age, systolic blood pressure, HDL cholesterol, LDL cholesterol, eGFR, and HbA1c levels. Furthermore, a significant positive correlation was also observed between ASCVD risk and serum UA levels (r = 0.34, p < 0.001; Table 4 and Figure 1).

**Table 4.** Correlation Analysis between 10-Year ASCVD Risk and Clinical Parameters.

	r	p
	
Age	0.76	<0.001
BMI	0.24	<0.001
Waist circumference	0.37	<0.001
Systolic BP	0.47	<0.001
Hemoglobin	0.40	<0.001
Total cholesterol	-0.01	0.406
Triglyceride	0.25	<0.001
HDL cholesterol	-0.39	<0.001
LDL cholesterol	0.13	<0.001
Serum creatinine	0.27	<0.001
eGFR	-0.32	<0.001
Uric Acid	0.34	<0.001
Fasting blood glucose	0.40	<0.001
HbA1c	0.38	<0.001

BMI: body mass index; BP: blood pressure; eGFR: estimated glomerular filtration rate; HbA1c: hemoglobin A1c; HDL: high-density lipoprotein; LDL: low-density lipoprotein.

To further investigate the independent association between serum UA levels and ASCVD risk, we performed multiple linear regression analyses. After adjusting for age, systolic blood pressure, hemoglobin, LDL cholesterol, eGFR, HbA1c, drinking, and smoking status, serum UA levels remained an independent predictor of ASCVD risk (β = 0.02, p < 0.001 in Table 5). In addition, we performed sex-stratified multiple linear regression analyses. After adjusting for the variables, serum UA levels remained an independent predictor of ASCVD risk in both males (β = 0.02, p < 0.001) and females (β = 0.03, p < 0.001) (Table 5).

**Table 5.** Multivariable Linear Regression Analysis of Serum Uric Acid Association with 10-Year ASCVD Risk.

	Overall		Male		Female	
	r = 0.97	p < 0.001	r = 0.96	p < 0.001	r = 0.96	p < 0.001
	β	p	β	p	β	p
Uric acid, mg/dL	0.02	<0.001	0.02	0.006	0.03	<0.001
Age, years	0.67	<0.001	0.86	<0.001	0.79	<0.001
Sex, male	-0.47	<0.001
Current drinker	-0.03	<0.001	-0.04	<0.001	-0.03	<0.001
Current smoker	0.13	<0.001	0.19	<0.001	0.08	<0.001
Systolic BP, mmHg	0.15	<0.001	0.17	<0.001	0.19	<0.001
Hemoglobin, g/dL	0.01	0.016	0.03	<0.001	-0.01	0.195
LDL cholesterol, mg/dL	0.14	<0.001	0.16	<0.001	0.18	<0.001
eGFR, mL/min/1.73m^2^	-0.01	0.151	-0.02	<0.001	0.02	<0.001
HbA1c, %	0.12	<0.001	0.15	<0.001	0.11	<0.001

BP: blood pressure; eGFR: estimated glomerular filtration rate; HbA1c: hemoglobin A1c; LDL: low-density lipoprotein.

## Discussion

In the present study, we showed a significant positive correlation between serum UA levels and 10-year ASCVD risk scores in the Japanese population who underwent regular health check-ups. Furthermore, multivariate analysis showed that serum UA levels were an independent predictor of 10-year ASCVD risk, even after adjustment for traditional risk factors. These findings are consistent with previous studies conducted in other Asian populations, such as the China-PAR (Prediction for ASCVD Risk in China) 10-year ASCVD risk scores ^(19), (20), (21), (22)^. On the other hand, a study by Tian et al. ^(23)^ has reported U-shaped or J-shaped associations between these factors. In Korea, another Asian country, the Korean Atherosclerotic Cardiovascular Disease Risk Prediction (K-CVD) model has been developed ^(24)^, although no studies have yet evaluated the association between serum UA levels and 10-year ASCVD risk using the K-CVD model. At present, there is no unified consensus on the relationship between serum UA levels and ASCVD risk even among Asian populations. This lack of consensus highlights the need to investigate the relationship in the Japanese population.

Our results also suggest a novel insight into the specific role of UA as an ASCVD risk marker in the Japanese population, given the distinctive cardiovascular disease pattern observed in the population, characterized by a lower incidence of coronary artery disease and a higher incidence of stroke compared to Western populations ^(6), (7), (8)^. In fact, UA has been reported to be involved in oxidative stress induction ^(25)^, inflammation promotion ^(7)^, vascular remodeling ^(26)^, endothelial dysfunction ^(27)^, and lipid metabolism alteration ^(28)^. On the other hand, the standardized regression coefficient β value for serum UA levels is relatively small in multivariate analysis (Table 5); therefore, its contribution to ASCVD development may be smaller than previously established risk factors.

Taken together, serum UA levels can play an important role in disease development by acting additively or synergistically with other risk factors in the Japanese population, although further studies are needed to identify the issues.

This study has several limitations. First, its retrospective cohort design precludes establishing a causal relationship between serum UA levels and ASCVD risk. Second, our cohort was composed of health check-up participants, who were relatively health-conscious individuals, and therefore ASCVD risk scores were potentially underestimated in this study compared to the scores in high-risk populations who did not receive health check-ups. Furthermore, the regional characteristics of the area where our hospital is located might have affected the results. Third, the actual predictive ability of serum UA levels was not investigated because our study did not employ ASCVD development as an endpoint. Fourth, our study did not include the information on antihypertensive agents, lipid-lowering therapy or UA-lowering therapy such as allopurinol or febuxostat, because we did not have data on these treatments. Similarly, data on precise purine intake were also unavailable. Fifth, we did not exclude the patients with gout or those with extremely high serum UA levels.

### Conclusions

This study indicates that serum UA levels may serve as a potential marker for ASCVD risk assessment in the Japanese population, although it is not yet an established clinical predictor. The results also suggest that serum UA measurement could contribute to improving ASCVD prevention strategies tailored to this population. However, further studies with hard cardiovascular endpoints are required to validate the clinical utility of UA measurement in ASCVD risk stratification.

## Article Information

### Conflicts of Interest

None

### Author Contributions

Conceived ideas: Takashi Matsuyama, Ryuichi Furuya, Hirotaka Fukasawa. Collected the data: Ryuichi Furuya. Analyzed the data: Takashi Matsuyama. Writing: Takashi Matsuyama, Hirotaka Fukasawa. All authors critically revised the manuscript and approved the final version.

### Approval by Institutional Review Board (IRB)

This study was approved by the Ethics Committee of Iwata City Hospital (Approval Code: 2024-003).
